# A Photoresponsive Artificial Viral Capsid Self-Assembled from an Azobenzene-Containing *β*-Annulus Peptide

**DOI:** 10.3390/ijms22084028

**Published:** 2021-04-14

**Authors:** Kazunori Matsuura, Seiya Fujita

**Affiliations:** 1 Department of Chemistry and Biotechnology, Graduate School of Engineering, Tottori University, Tottori 680-8552, Japan; fujita.tottori1208@gmail.com; 2 Centre for Research on Green Sustainable Chemistry, Tottori University, Tottori 680-8552, Japan

**Keywords:** self-assembly, *β*-annulus peptide, azobenzene, photoisomerization, capsid, guest-molecule release

## Abstract

Photoinduced structural changes in peptides can dynamically control the formation and dissociation of supramolecular peptide materials. However, the existence of photoresponsive viral capsids in nature remains unknown. In this study, we constructed an artificial viral capsid possessing a photochromic azobenzene moiety on the peptide backbone. An azobenzene-containing *β*-annulus peptide derived from the tomato bushy stunt virus was prepared through solid-phase synthesis using Fmoc-3-[(3-aminomethyl)-phenylazo]phenylacetic acid. The azobenzene-containing *β*-annulus (*β*-Annulus-Azo) peptide showed a reversible trans/cis isomerization property. The *β*-annulus-azo peptide self-assembled at 25 μM into capsids with the diameters of 30–50 nm before UV irradiation (trans-form rich), whereas micrometer-sized aggregates were formed after UV irradiation (cis-form rich). The artificial viral capsid possessing azobenzene facilitated the encapsulation of fluorescent-labeled dextrans and their photoinduced release from the capsid.

## 1. Introduction

Natural viral capsids are attractive organic materials with discrete sizes and hollow interior nanospaces, which are applied as nanocarriers for drug delivery, nanoreactors, and vaccine platforms [1,2,3,4,5]. Exogenous stimuli responsiveness has been recognized as a promising property beneficial for controlled drug delivery [6]. Although most viral capsids have static and stable capsule structures, only a few stimuli-responsive viral capsids are known. For example, the cowpea chlorotic mottle virus (CCMV) capsid shows pH-responsive swelling behavior attributed to its significant conformational changes [7,8]. The pH-responsive structural change enables the control of the encapsulation of proteins in the CCMV capsid [9]. The hibiscus chlorotic ringspot Carmovirus capsid shows structural changes in response to calcium ions, which enables the control of the release of anticancer drugs in response to calcium ions [10]. Notably, to date, the existence of photoresponsive viral capsids in nature remains unverified.

Photoresponsive organic materials undergo various structural changes upon photoirradiation under controlled location, timing, light wavelength, and strength. The introduction of photochromic groups to peptides and proteins enables the spatiotemporal control of structure and function upon light irradiation [11,12]. For example, Hilvert et al. demonstrated that a *β*-hairpin peptide possessing photochromic azobenzene showed dramatic structural changes caused by photoisomerization to form aggregates [13,14]. Nilsen et al. succeeded in photocontrolling the fibril formation of amyloid-*β* using an azobenzene-containing peptide [15].

**Figure 1:**
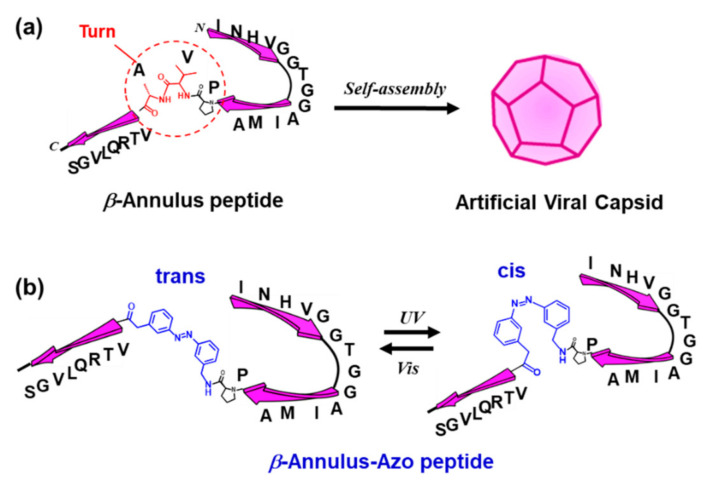
(**a**) Formation of an artificial viral capsid by the self-assembly of *β*-annulus peptides and (**b**) the photoisomerization of the azobenzene-containing *β*-annulus (*β*-annulus-azo) peptide.

The capsid of the tomato bushy stunt virus (TBSV) self-assembles from 180 quasi-equivalent protein subunits, where the *β*-annulus motif participates in the formation of a dodecahedral internal skeleton [16,17]. Previously, we developed a hollow artificial viral capsid with a size of 30–50 nm self-assembled from the 24-mer *β*-annulus peptides (INHVGGTGGAIMAPVAVTRQLVGS) of TBSV [18,19]. The encapsulation of various guest molecules, such as nucleic acids and proteins, in the artificial viral capsid was achieved via electrostatic interactions or the N-terminal modification of the *β*-annulus peptide [19,20,21,22,23,24]. Furthermore, the modification of the C-terminal, which is expected to be directed toward the exterior of the capsid, enabled the exterior modification of capsids with various functional molecules [19,25,26,27,28,29,30]. However, the construction of artificial viral capsids that can bedynamically control the formation and dissociation in response to external stimuli remains unexplored. In the present study, we developed an artificial viral capsid possessing a photochromic azobenzene moiety on the peptide backbone for controlling the self-assembly and release of guest molecules from the capsid through light irradiation (Figure 1).

## 2. Results

### 2.1. Synthesis and Photoisomerization of Azobenzene-Containing β-Annulus Peptide

**Figure 2:**
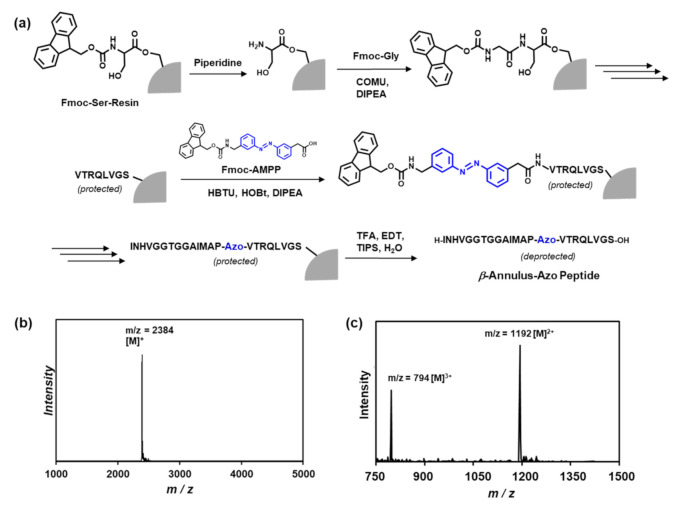
(**a**) Solid-phase synthesis of the *β*-annulus-azo peptide, (**b**) matrix-assisted laser desorption ionization-time of flight mass spectrometry (MALDI-TOF) mass spectrum (matrix: α-CHCA), and (**c**) the electrospray ionization-time of flight mass spectrometry (ESI-TOF)-mass spectrum (in methanol, positive mode) of the purified *β*-annulus-azo peptide.

The Pro14-Val15-Ala16 sequence in the *β*-annulus peptide, which forms a turn structure in TBSV [16,17], might play a pivotal role in the formation of the artificial viral capsid. Thus, we designed an azobenzene-containing *β*-annulus (*β*-annulus-azo) peptide, of which Val15 and Ala16 were substituted by 3-[(3-aminomethyl)-phenylazo]phenylacetic acid (AMPP) to construct a photoresponsive artificial capsid (Figure 1). Fmoc-AMPP was synthesized following the procedures in the literature [13,14]. The *β*-annulus-azo peptide (INHVGGTGGAIMAP-*Azo*-VTRQLVGS) was synthesized using the standard Fmoc-protected solid-phase method, which was purified through reversed-phase high-performance liquid chromatography (HPLC) and confirmed through the matrix-assisted laser desorption ionization-time of flight mass spectrometry (MALDI-TOF MS) and electrospray ionization mass spectrometry (ESI-MS) (Figure 2).

**Figure 3:**
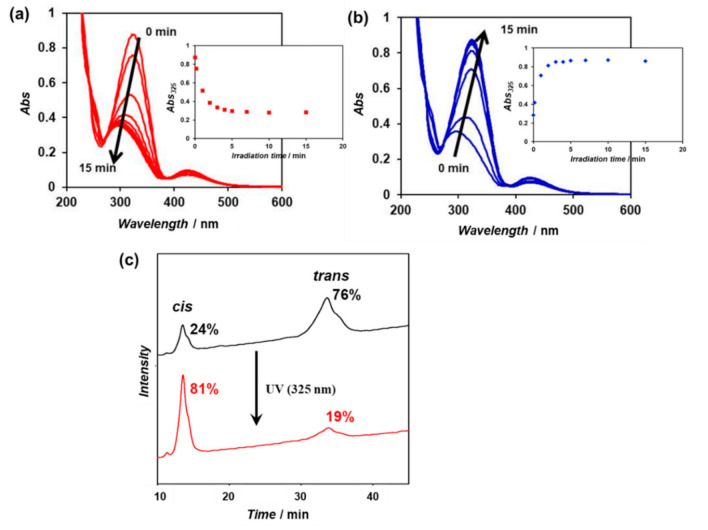
(**a**,**b**) Time-dependent UV–vis spectra of the *β*-annulus-azo peptide (100 μM) in water under (**a**) UV-light (325 nm) and (**b**) visible-light (425 nm) irradiations at 25 °C; (**c**) the reversed-phase high-performance liquid chromatography (HPLC) charts of the *β*-annulus-azo peptide eluted with a linear gradient of CH_3_CN/water (26/74 to 28/72 for over 100 min), containing 0.1% trifluoroacetic acid (TFA) before and after UV-light irradiation for 15 min.

An aqueous solution (100 μM) of the purified *β*-annulus-azo peptide was irradiated with visible light (425 nm) for 30 min to enrich the trans form, followed by UV irradiation (325 nm) for 15 min at 25 °C. The time-dependent UV–vis spectra of the aqueous solution showed a decrease in the characteristic strong π–π* transition band between 290 and 380 nm within 15 min, indicating the achievement of the cis-enriched photostationary state (Figure 3a). The subsequent visible-light irradiation (425 nm) of the cis-rich peptide solution induced the recovery to the trans-rich state within 15 min (Figure 3b). These results indicate that the *β*-annulus-azo peptide is capable of reversible photoisomerization in aqueous solutions. The cis/trans ratios of the trans-rich and cis-rich peptide solutions (before and after UV irradiation) were estimated from the relative peak area of the reversed-phase HPLC charts to be 24/76 and 81/19, respectively (Figure 3c).

### 2.2. Photoresponsive Self-Assembly Behavior of the β-Annulus-Azo Peptide

**Figure 4:**
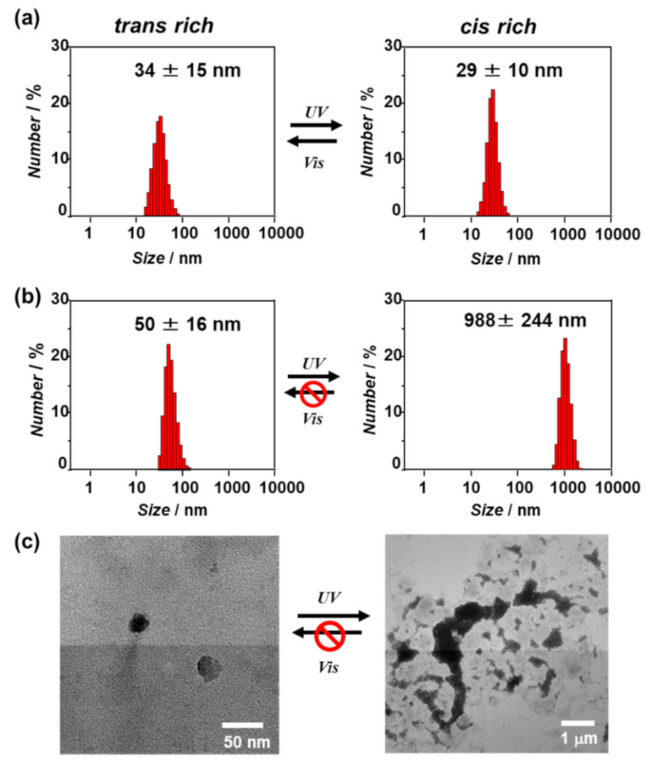
(**a**,**b**) Dynamic light scattering (DLS) size distributions of the aqueous solutions of the *β*-annulus-azo peptide (**a**: 100 μM, **b**: 25 μM) after UV and visible-light irradiations for 15 min at 25 °C; (**c**) the TEM images of the aqueous solutions of the *β*-annulus-azo peptide (25 μM) after UV and visible-light irradiations for 15 min at 25 °C. The samples were stained with 2% aq. phosphotungstic acid.

The self-assembly behavior of the *β*-annulus-azo peptide in water after UV or visible-light irradiation was evaluated through dynamic light scattering (DLS) measurement and transmission electron microscopy (TEM) observation (Figure 4). At 100 μM, the DLS size-distribution showed that the *β*-annulus-azo peptide formed assemblies with a size of approximately 30 nm after both UV (cis-form rich) and visible-light (trans-form rich) irradiations, suggesting the formation of stable capsid structures regardless of photoisomerizaton (Figure 4a, these TEM images are shown in Figure S1). In contrast, the DLS size-distribution of the *β*-annulus-azo peptide at 25 μM showed a significant increase from 50 to 988 nm upon UV irradiation (Figure 4b). Moreover, the TEM results showed the formation of spherical artificial viral capsids with the sizes of 30–40 nm before UV irradiation (trans-form rich), whereas micrometer-sized aggregates were formed after UV irradiation (cis-form rich) (Figure 4c). The micrometer-sized aggregates barely reverted to the capsid structure even after visible-light reirradiation. Thus, the *β*-annulus-azo peptide at 25 μM showed an irreversible change of the assembled structures by photoisomerization. The difference in the photoresponsiveness of the *β*-annulus-azo peptides between 100 and 25 μM might be ascribed to the dynamic stability of the assemblies. The critical aggregation concentration (CAC) of the *β*-annulus-azo peptide was estimated by the concentration dependence on the scattering intensity to be approximately 10 μM (Figure S2). In general, there are dynamic equilibrium between assembly and dissociation at the concentration near CAC. Thus, it is reasonable that the peptide is probably susceptible to dynamic structural changes at 25 μM. CD spectrum before UV irradiation showed a negative peak at 197 nm and a negative shoulder peak at 218 nm (Figure S3), indicating coexistence of random coil and *β*-structures, which is similar to the secondary structure of original 24-mer *β*-annulus peptide [18]. The negative molar ellipticity increased after UV irradiation, indicating the increase of contents of random coil by photoisomerization of the azobenzene moiety from the trans to the cis form.

### 2.3. Photoresponsive Release of Guest Molecules from the Artificial Viral Capsid

Fluorescence correlation spectroscopy (FCS) can analyze spontaneous fluorescence intensity fluctuations in a microscopic detection volume of approximately 10 fL to estimate the diffusion time and size of fluorescence molecules [31,32]. Previously, we reported the FCS analysis of the encapsulation of quantum dots in artificial viral capsids via electrostatic interactions [23]. Herein, we evaluated the encapsulation of fluorescein isothiocyanate (FITC)-labeled dextran (70 kDa) as a guest molecule in the artificial viral capsid possessing azobenzene, as well as its photoresponsive release from the capsid, using FCS. The FITC-labeled dextran encapsulated in the artificial viral capsids was expected to exhibit a relatively slow autocorrelation function decay due to its larger apparent particle size compared to that of the free dextran.

**Figure 5:**
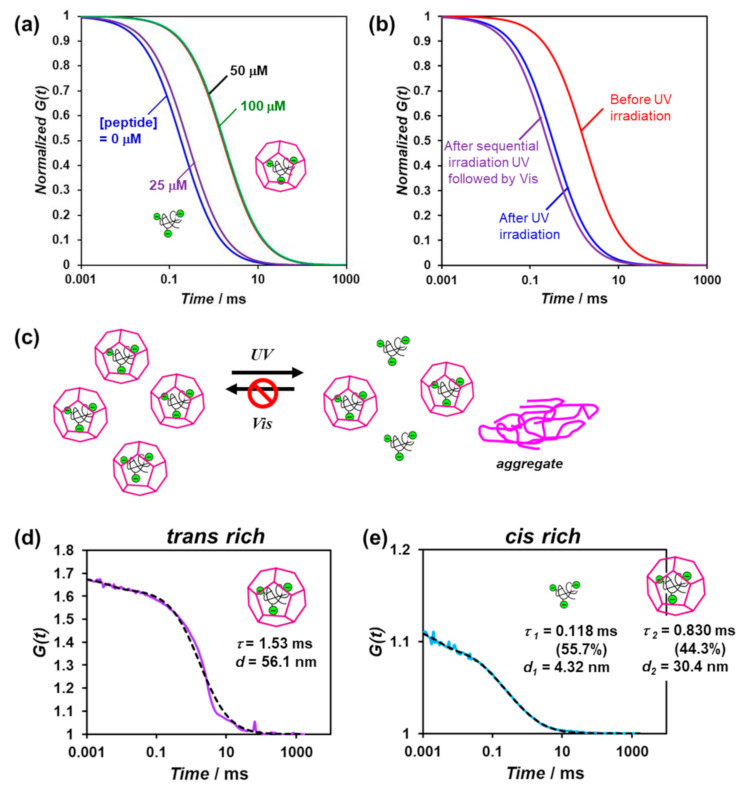
(**a**) Normalized autocorrelation curves of the mixture of the 70-kDa FITC-labeled dextran (0.1 μM) and *β*-annulus-azo peptide (0−100 μM) in water at 25 °C; (**b**) the normalized autocorrelation curves of the mixture of the 70-kDa FITC-labeled dextran (0.1 μM) and *β*-annulus-azo peptide (50 μM) before and after photoirradiation in water at 25 °C; (**c**) schematic illustration of the photocontrolled release of the FITC-labeled dextran from the artificial viral capsids; (**d**,**e**) measured (solid) and fitted (dot) autocorrelation curves for the mixture of the 70-kDa FITC-labeled dextran (0.1 μM) and *β*-annulus-azo peptide (50 μM) before (**d**) and after (**e**) UV-light irradiation in water at 25 °C. These mixtures were prepared by adding was dissolved in an aqueous solution of 0.1-μM FITC-labeled dextran to the lyophilized powder of the trans-rich *β*-annulus-azo peptide to ensure encapsulation.

The lyophilized powder of the trans-rich *β*-annulus-azo peptide was dissolved in an aqueous solution of 0.1-μM FITC-labeled dextran (70 kDa), after which the mixture was incubated for 1 h at 25 °C prior to FCS analysis. Figure 5a shows the normalized autocorrelation function curves derived from the FITC-labeled dextran in the presence of the various concentrations of the *β*-annulus-azo peptide. At concentrations above 50 μM, the autocorrelation function curve shifted toward longer diffusion times, indicating the encapsulation of the FITC-labeled dextrans in the artificial viral capsids. The autocorrelation function curve was fitted to a single component model with the diffusion time of 1.53 ms (Figure 5d). The apparent hydrodynamic diameter was calculated to be 56.1 nm using Stokes–Einstein equation, which is comparable to the sizes of the artificial viral capsids. When an aqueous solution of FITC-labeled dextran was added to an aqueous solution of the *β*-annulus-azo peptide, autocorrelation function curve was fitted to a single component model with the diffusion time of 0.196 ms (Figure S4), suggesting the existence of only free FITC-labeled dextrans. This indicates that the longer diffusion time was derived from the encapsulation of FITC-labeled dextran in capsid, not from a simple interaction between *β*-annulus-azo peptide and FITC-labeled dextran. 

When UV-light irradiation was applied to the FITC-labeled dextran encapsulated in the artificial viral capsids comprising 50-μM trans-rich *β*-annulus-azo peptide, the autocorrelation function curve shifted toward shorter diffusion times (Figure 5b). Based on the results of the dual-component curve fitting, we estimated the diffusion time and ratio to be 0.118 ms and 55.7% for the fast component and 0.830 ms and 44.3% for the slow component, respectively (Figure 5e). This suggests the coexistence of the free FITC-labeled dextrans (55.7%) and those encapsulated in the artificial viral capsids (44.3%). Thus, a portion of the FITC-labeled dextrans was released from the capsid via the photoisomerization of the *β*-annulus-azo peptide. However, after sequential UV and visible-light irradiations, the re-encapsulation of the FITC-labeled dextrans in the capsid was not indicated (Figure 5b,c), probably due to the formation of the aggregates of the cis-rich *β*-annulus-azo peptide, as shown in Figure 4.

## 3. Discussion

Previously, we reported that 24-mer *β*-annulus peptides of TBSV spontaneously self-assembled into artificial viral capsids with sizes of 30–50 nm in water [18]. The artificial viral capsids can be constructed by simply dissolved the lyophilized powder of peptide in deionized water without sonication or heating. The synchrotron small angle X-ray scattering (SAXS) profile of artificial viral capsid indicated the existence of a hollow inside of the particle [18]. We estimated that the Pro14-Val15-Ala16 sequence in the *β*-annulus peptide, which forms a turn structure in TBSV [16,17], might play a pivotal role in the formation of the artificial viral capsid. In fact, *β*-annulus peptide in which Pro is replaced with Ala (INHVGGTGGAIMA**A**VAVTRQLVGS) self-assembled into fibrous structures, not capsids (Figure S5). Thus, we designed the azobenzene-containing *β*-annulus (*β*-annulus-azo) peptide, of which Val15 and Ala16 were substituted by AMPP to show a reversible trans/cis isomerization property. The *β*-annulus-azo peptide self-assembled into artificial viral capsids with the diameters of 30–50 nm before UV irradiation (trans-form rich). Although we attempted to measure SAXS profile of aqueous solutions of the *β*-annulus-azo peptide assembly, unfortunately, we could not obtain any clear SAXS profile probably due to less uniformity of the assemblies. Since the size distribution of *β*-annulus-azo peptide assembly is comparable to the original artificial viral capsid (30–50 nm), it is probable that the assembly also has a hollow inside rather than a micelle structure. 

At 100 μM, the *β*-annulus-azo peptide formed assemblies with the size of approximately 30 nm after both UV (cis-form rich) and visible-light (trans-form rich) irradiations, suggesting the formation of stable capsid structures regardless of photoisomerizaton. Contrarily, at 25 μM near the CAC (10 μM), the *β*-annulus-azo peptide showed an irreversible change of the assembled structures by photoisomerization, i.e., it formed the capsids of 30–40-nm sizes before UV irradiation (trans-form rich), whereas it changed to micrometer-sized aggregates after UV irradiation (cis-form rich). The irreversible structural change of the capsids might be caused by the dynamic structural changes near the CAC. The CAC of the *β*-annulus-azo peptide is slightly lower than that of 24-mer original *β*-annulus peptide (25 μM) [18]. The stability of artificial viral capsid, which is estimated by CAC, can be controlled by chemical modifications of *β*-annulus peptide. For example, modification of Ni-NTA to N-terminal of *β*-annulus peptide significantly decreased the CAC (0.053 μM) [24]. Decoration of human serum albumin on artificial viral capsid furtherly decreased the CAC (0.01 μM) [28].

Furthermore, we demonstrated, by FCS measurements, that the FITC-labeled dextrans were encapsulated in the artificial viral capsids possessing azobenzene and released by photoisomerization. This proof-of-concept will facilitate the development of a photoinduced drug-release system using artificial viral capsids, which can be applied for novel materials in drug delivery systems. Although attention should be paid to the limitation of the photoinduced irreversible structural change of the capsids, we attempt to improve the molecular design of peptides possessing photochromic dye groups.

## 4. Materials and Methods

### 4.1. General

Fmoc-3-[(3-aminomethyl)-phenylazo]phenylacetic acid (Fmoc-AMPP) was synthesized following a procedure reported in the literature [13,14] and confirmed through ^1^H NMR spectroscopy. The 70-kDa FITC-labeled dextran (FITC:glucose = 1:250) was purchased from Sigma-Aldrich. Other reagents were obtained from commercial sources and were used without further purification. High-resistivity ultrapure water (>18 MΩ cm), which was purified using a Millipore purification system (Milli-Q water), was used as a solvent for the peptides. Reversed-phase HPLC was performed at ambient temperature using a Shimadzu LC-6AD liquid chromatography system equipped with Inertsil ODS-3 (GL Science, Torrance, CA, USA) columns (250 × 4.6 or 250 × 20 mm^2^) and a UV–vis detector (220 nm, Shimadzu SPD-10AVvp, Kyoto, Japan). The MALDI-TOF mass spectra were obtained using an Autoflex T2 instrument (Bruker Daltonics, Billerica, MA, USA) in the linear/positive mode, with a matrix (α-cyano-4-hydroxy cinnamic acid: α-CHCA). The ESI mass spectra were obtained using a Q Exactive™ Focus quadrupole/orbitrap hybrid mass spectrometer (Thermo Fisher Scientific, Waltham, MA, USA) in the positive mode in a methanol solution.

### 4.2. Synthesis of β-Annulus-Azo Peptide

The β-annulus peptide containing azobenzene (H-INHVGGTGGAIMAP-Azo-VTRQLVGS-OH) was synthesized at the 0.125-mmoL scale on a commercially available Fmoc-Ser(tBu)-Alko-PEG resin (0.24 mmoL/g, Watanabe Chemical Ind. Ltd., Hiroshima, Japan) using microwave-assisted Fmoc-based coupling reactions. The Fmoc group was deprotected using 20% piperidine in N,N-dimethylformamide (DMF) at room temperature for 30 min. The Fmoc-protected amino acids (4 equiv.), except for Fmoc-AMPP, were coupled using (1-cyano-2-ethoxy-2-oxoethylidenaminooxy) dimethylamino-morpholino-carbenium hexafluorophosphate (COMU, 4 equiv.) in N-methylpyrrolidone (NMP) as the activating agent and DIPEA (8 equiv.) in NMP as the base. Because Fmoc-AMPP was precipitated with COMU in NMP, the coupling of Fmoc-AMPP was conducted using 1-[bis(dimethylamino)methylene]-1H-benzotriazolium 3-oxide hexafluorophosphate (HBTU, 4 equiv.), 1-hydroxybenzotriazole monohydrate (HOBt▪H_2_O, 4 equiv.), and DIPEA (8 equiv.) in NMP. The coupling reactions were conducted using a microwave synthesizer (Biotage Initiator+) for 5 min at 35 W at the maximum temperature of 75 °C throughout the synthesis. The progression of the coupling reaction and Fmoc deprotection was confirmed using the TNBS test kit (Tokyo Chemical Industry Co., Ltd., Kyoto, Tokyo). After the loading of the amino acids, the peptide was deprotected and cleaved from the resin through treatment with a cleavage cocktail of trifluoroacetic acid (TFA)/water/triisopropylsilane (9.5/0.25/0.25 in mL, respectively) at room temperature for 40 min. The reaction mixtures were filtered to remove the resins, and the filtrates were concentrated under a vacuum. The peptide was precipitated by adding methyl tert-butyl ether (MTBE) to the residue, and the supernatant was decanted. After three washes with MTBE, the crude peptide was lyophilized to obtain a flocculent solid (yield: 45.4%). The reversed-phase HPLC of the crude peptide eluted with a linear gradient of CH_3_CN/water containing 0.1% TFA (27/73 to 29/71 over 100 min) showed two peaks at 13 min (cis form) and 33 min (trans form), respectively. The isolated yield was 6.0%. MALDI-TOF MS (matrix: α-CHCA): m/z = 2384 [M]^+^; ESI-MS (methanol): m/z = 1192 [M]^2+^, 794 [M]^3+^.

### 4.3. Photoisomerization of the β-Annulus-Azo Peptide

The purified *β*-annulus-azo peptide was dissolved in ultrapure water to prepare a 100-μM aqueous solution. The 150-W Xe lamp of a JASCO FP-8200 spectrophotometer was used as the light source for the photoisomerization of azobenzenes in an ultramicro quartz cell (optical path length: 10 mm, volume: 50 μL). Initially, the aqueous solution of the *β*-annulus-azo peptide was irradiated with 425-nm monochromic light for 30 min at 25 °C, followed by 325-nm monochromic light for 15 min at 25 °C, and finally 425-nm monochromic light for 15 min at 25 °C. During the photoirradiation, the time-dependent UV–vis spectra were recorded at 25 °C in the ultramicro quartz cell using a JASCO V-630 spectrophotometer equipped with a Peltier-type thermostatic cell holder. The cis/trans ratio of the *β*-annulus-azo peptide was determined by the relative peak area obtained from the reversed-phase HPLC charts of the peptide eluted with a linear gradient of CH_3_CN/water (26/74 to 28/72 for over 100 min), containing 0.1% TFA before and after UV-light irradiation for 15 min.

### 4.4. Self-Assembling Behavior of the β-Annulus-Azo Peptide

An aqueous solution of the *β*-annulus-azo peptide (100 μM) was irradiated with 425- or 325-nm monochromic light for 15 min in an ultramicro quartz cell at 25 °C. After the solution was diluted with ultrapure water, DLS measurement was performed using a Zetasizer Nano ZS (MALVERN, Worcestershire, UK) instrument with an incident He–Ne laser (633 nm) at 25 °C. The G(τ) (the correlation times of the scattered light intensities) was measured several times, after which the diffusion coefficient was calculated from the means of the G(τ) values. Using the Stokes–Einstein equation, the hydrodynamic diameters of the scattering particles were calculated. The aliquots (5 μL) of the photoirradiated aqueous solutions were applied to C-SMART Hydrophilic TEM grids (ALLIANCE Biosystems Inc) for 1 min, after which the droplet was removed using a filter paper. The TEM grids were placed into a staining aqueous solution, 2% phosphotungstic acid (5 μL), for 1 min and subsequently removed. After the sample-loaded TEM grids were dried in vacuo, they were analyzed through TEM (JEM 1400 Plus; JEOL, Tokyo, Japan) at an accelerating voltage of 80 kV.

### 4.5. Photocontrolled Release of the FITC-Labeled Dextran from the Artificial Viral Capsid

An aqueous solution of the *β*-annulus-azo peptide (100 μM) was photoirradiated at 1.0 mW/cm^2^ for 1 h using SAN-EI Super Cure-204S C-type (SAN-EI Electronic Inc., Osaka, Japan) through a 435-nm narrow-band-pass filter. After the trans-rich *β*-annulus-azo peptide was lyophilized, the powder was dissolved in an aqueous solution of the 0.1-μM FITC-labeled dextran (70 kDa), after which the mixture was incubated for 1 h at 25 °C. The FCS analysis of the solution was conducted on an FCS Compact BL (Hamamatsu Photonics KK, Hamamatsu, Japan) in a microwell slide (25 μL) using a 473-nm laser at 25 °C. Subsequently, the solution was photoirradiated at 1.0 mW/cm^2^ for 30 min through a 365-nm narrow-band-pass filter, incubated for 10 min at 25 °C, and subsequently subjected to FCS analysis. The diffusion time (*τ*) and ratio (*y*) of the fast and slow components were obtained by curve fitting the autocorrelation function *G(t)* obtained from the FCS measurements according to Equation (1):

(1) $$G\left(t\right)=1+\frac{1}{N}\times \left(\frac{y_{1}}{\left(1+\frac{t}{\tau_{1}}\right)\sqrt{1+\frac{1}{k^{2}}\cdot \frac{t}{\tau_{1}}}}+\frac{y_{2}}{\left(1+\frac{t}{\tau_{2}}\right)\sqrt{1+\frac{1}{k^{2}}\cdot \frac{t}{\tau_{2}}}}\right)$$

where *N* is the average number of FITCs in the detection area and *K* is the structural parameter. The hydrodynamic radius (*r*) of the FITC-labeled dextran was calculated using Equations (2) and (3):

(2) $$\tau =\frac{\omega^{2}}{4D}$$

(3) $$D=\frac{k_{B}T}{6\pi \eta r}$$

where τ is the diffusion time of the FITC-labeled dextran in the detection area, ω is the radius of the detection area evaluated through reference measurement using Alexa 488, T is the absolute temperature, k_B_ is the Boltzmann constant, η is the viscosity of the solvent, and D is the diffusion coefficient of the FITC-labeled dextran.

## Data Availability

Not applicable.

## Supplementary Materials

Supplementary materials can be found at https://www.mdpi.com/article/10.3390/ijms22084028/s1.
