# Supplementary material for: A Photoresponsive Artificial Viral Capsid Self-Assembled from an Azobenzene-Containing β-Annulus Peptide
Source: Int J Mol Sci. 2021 Apr 14;22(8):4028. doi: 10.3390/ijms22084028 (PMC8070682; doi:10.3390/ijms22084028)
Supplement: Supplementary file 1 [file ijms-22-04028-s001.pdf]

Supplementary Materials

Photo-responsive Artificial Viral Capsid self-assembled from Azobenzene-containing *β*-Annulus Peptide

Kazunori Matsuura [1,2]*, Seiya Fujita [1]

[1] Department of Chemistry and Biotechnology, Graduate School of Engineering, Tottori University, Tottori 680-8552, Japan.

[2] Centre for Research on Green Sustainable Chemistry, Tottori University, Tottori 680-8552, Japan.

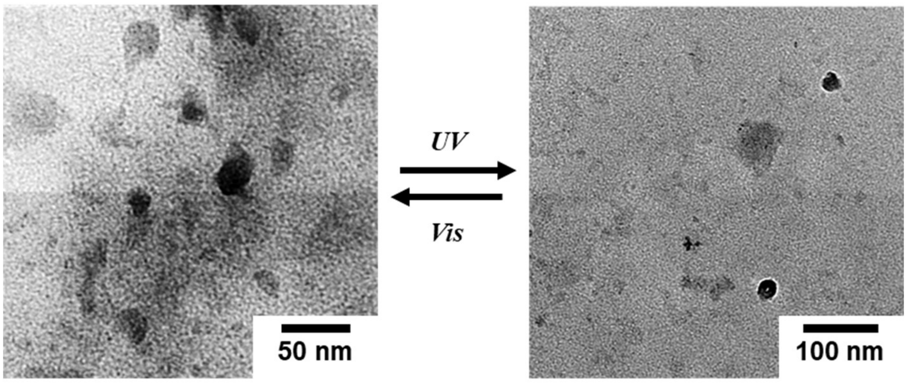

Figure S1. TEM images for aqueous solutions of *β*-annulus-azo peptide (100 μM) after UV and visible light irradiation for 15 min at 25°C. The samples were stained with 2% phosphotungstic acid aq.

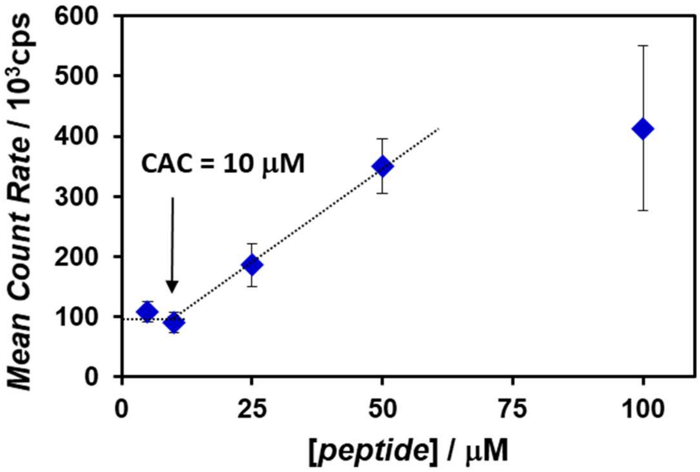

Figure S2. Effect of concentration of the *β*-annulus-azo peptide on scattering intensity determined by DLS at 25°C in water.

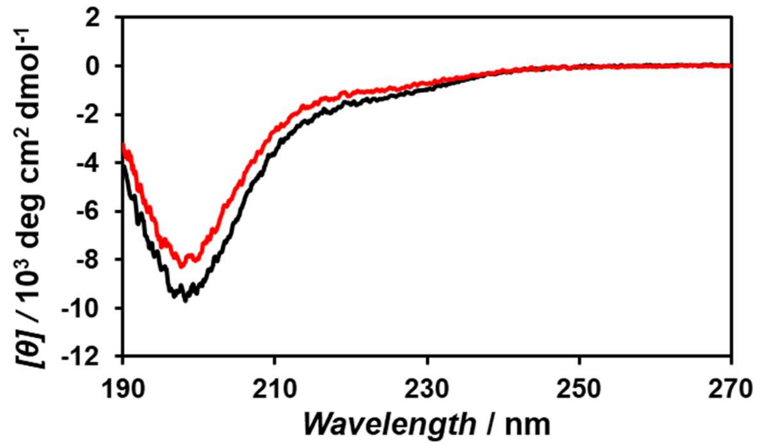

Figure S3. CD spectra of the aqueous solutions of the β-annulus-azo peptide before (red) and after (black) UV irradiation for 15 min at 25°C in water.

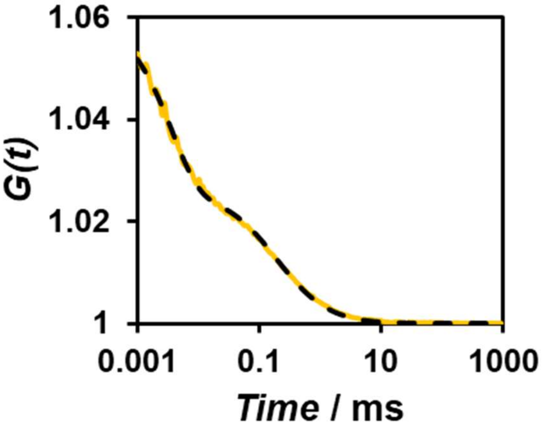

Figure S4. Measured (solid) and fitted (dot) autocorrelation curves for the 70-kDa FITC-labeled dextran (0.1 μM) mixed with an aqueous solution of the β-annulus-azo peptide (50 μM) at 25°C.

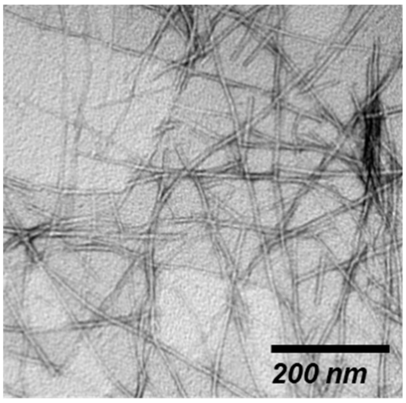

Figure S5. TEM images for aqueous solutions (100 μM) of *β*-annulus peptide in which Pro is replaced with Ala (INHVGGTGGAIMA**A**VAVTRQLVGS) at 25°C. The samples were stained with 2% phosphotungstic acid aq.
